# The impact of antibiotic treatment for syphilis, chlamydia, and gonorrhoea during pregnancy on birth outcomes: A systematic review and meta-analysis

**DOI:** 10.7189/jogh.13.04058

**Published:** 2023-06-16

**Authors:** Hannah Tong, Austin Heuer, Neff Walker

**Affiliations:** Department of International Health, Bloomberg School of Public Health, Johns Hopkins University, Baltimore, Maryland, USA

## Abstract

**Background:**

Sexually transmissible infections are important causes of loss of health and lives in women and infants worldwide. This paper presents the methods and results of a systematic review that focuses on the impact of antibiotic treatment for syphilis, chlamydia, and gonorrhoea during pregnancy on birth outcomes for the Lives Saved Tool (LiST).

**Methods:**

We searched PubMed, Embase, Cochrane Libraries, Global Health and Global Index Medicus for articles available until May 23rd, 2022. The search criteria focused on the impact of treatment for the three sexually transmitted infection among pregnant women. Nearly all the articles found were non-randomized studies.

**Results:**

Treatment for pregnant women with active syphilis reduced the risk of preterm birth by 52% (95% CI = 42%-61%; 11 043 participants, 15 studies; low quality); stillbirth by 79% (95% CI = 65%-88%; 14 667 participants, eight studies; low quality); and low birth weight by 50% (95% CI = 41%-58%; 9778 participants, seven studies; moderate quality). Treatment for pregnant women with chlamydia infection reduced the risk of preterm birth by 42% (95% CI = 7%-64%; 5468 participants, seven studies; low quality) and might reduce the risk of low birth weight by 40% (95% CI = 0%-64%; 4684 participants, four studies; low quality). No studies provided data on treatment of gonorrhoea therefore no meta-analysis was conducted.

**Conclusions:**

Because few studies adjusted for potential confounding factors, the overall quality of evidence was considered low. However, given the consistent and large effects, we recommend updating the estimated effect of timely detection and treatment for syphilis on preterm birth and stillbirth in the LiST model. More research is required to ascertain the effect of antibiotic treatment for chlamydia and gonorrhoea infection in pregnancy.

Sexually transmissible infection (STI) in pregnancy is still prevalent worldwide. It was estimated that more than 340 million new cases of STIs occur throughout the world every year [1-3]. These STIs are important cause of loss of health and lives in women and infants [4,5]. Much research has focused on the testing and treatment of common curable STIs like syphilis, chlamydia, and gonorrhoea [6-9]. Diagnosis and treatment of syphilis has also been proven to be feasible, relatively inexpensive, and cost-effective in various settings [10]. However, the implementation of these effective interventions was not ideal. And curable sexually transmissible infections (STIs) in pregnancy still represents an opportunity to improve health outcomes of women and infants worldwide.

This paper will present a systematic review and meta-analysis of available data on the impact of treatment for syphilis, chlamydia and gonorrhoea among pregnant women and its impact on the risk of poor birth outcomes. The primary purpose of this review was to update and extend the assumptions about the impact of interventions to reduce sub-optimal birth outcomes (small for gestational age and preterm births) for the Lives Saved Tool (LiST). LiST is a widely used model that allows users to estimate the impact of scaling up intervention coverage or adding new interventions [11,12]. As part of the process of updating and expanding the model, recent work has focused on identifying potential risk factors and expanding the treatment interventions that could be provided to antepartum women or during antenatal care that could reduce the occurrence of poor birth outcomes. In the two recent review papers, we identified that syphilis, chlamydia, and gonorrhoea might be risk factors for SGA or PTB [13,14]. We did not identify any evidence of hepatitis B infection and poor birth outcomes. It is noted that HIV was also identified as a risk factor but treatment or interventions for HIV was beyond the scope of the LiST model. The other model – AIM, housed in the same software as LiST, estimates the impact of human immunodeficiency virus (HIV) / acquired immunodeficiency syndrome (AIDS) interventions.

Looking for evidence of efficacy of treatment for syphilis, chlamydia, and gonorrhoea among pregnant in reducing the risk of poor birth outcomes in low- and middle-income countries (LMICs) has some specific issues. One issue is that treatment for syphilis and to a lesser degree chlamydia and gonorrhoea is an established standard of care intervention, so it is unethical to run a standard treatment vs no treatment randomized trial. Therefore, data on efficacy will be based on observational and retrospective data. Using these data one can compare birth outcomes for those pregnant women who are infected and then treated or untreated with the differences in birth outcomes reflecting the effects of treatment. We hope that studies will present risk adjusted for confounding variables. We also will look for comparisons that look at differences in both timings (early vs late in pregnancy) of testing and treatment as well as adequacy of treatment.

A second issue this review faced is that pregnant women in low- and middle-income countries face a very different set of risk factors and exposure levels to many of these risk factors than do women in high-income countries [13,14]. This means that data on efficacy from high-income countries maybe be less applicable to the pregnant women in LMICs. This analysis presented here using data from countries of all income strata but quality of the estimate of efficacy is adjusted for this.

## METHODS

This reporting of this systematic review was guided by the standards of the Preferred Reporting Items of Systematic Review and Meta-Analysis (PRISMA) statement. We systematically reviewed the published literature to identify studies of treatment of curable sexually transmitted disease (STI) in pregnancy for the prevention of suboptimal birth outcomes. The search was conducted on May 23, 2022. We searched PubMed, EMABASE, Cochrane Libraries, Global Health, and Global Index Medicus and included publications in any language. Combination of the following search terms with the OR Boolean operator were used: “Chlamydia Infection” “Gonorrhea” “Syphilis” “Pregnancy” “Pregnant Women” “Pregnancy Trimesters” “Premature Birth” “Obstetric labor, Premature” “Infant, Premature” “Infant Mortality” “Infant, Low Birth Weight” “Infant, Very Low Birth Weight” “Fetal Growth Retardation” “Fetal Death” “Perinatal Death” “Perinatal Mortality”. The complete search strategies in the databases described above were included in Table S1 in the **Online Supplementary Document**.

### Inclusion / exclusion criteria

We used the PICO approach (Population, Intervention, Comparison, and Outcome) to identify the studies to be included. The population of interest is pregnant women with active syphilis, gonorrhoea, or chlamydia infection. The intervention being reviewed is any antibiotic treatment for syphilis, gonorrhoea, or chlamydia. The comparison group is pregnant women with active syphilis, gonorrhoea, or chlamydia who received no or inappropriate antibiotic treatment. The outcomes of interest are suboptimal birth outcomes including preterm births (less than 37 gestational weeks), stillbirth, low birthweight (less than 2500 grammes), perinatal deaths, any adverse pregnancy outcomes (APO), one or more of the outcomes listed before. Within this structure we considered both randomized control trials and observational studies, excluding studies that only used modelling to predict the outcomes. For duplicate reports of trials or studies we counted outcome data only once. Possible adverse effects of antibiotic treatment were not addressed as part of this review.

### Data collection and analysis

Two review authors independently reviewed the abstract of search results to determine the relevancy. For studies that were deemed relevant, two review authors independently reviewed the full texts to determine if they met the inclusion criteria for this review. Where there was disagreement, advice from a third review author was sought and consensus reached by discussion. A pilot data extraction form was developed, and all three reviewers tested using the extraction sheet and agreed on the final version of the form. All studies meeting the criteria above were abstracted onto the data abstraction form for each outcome of interest, including study title, country, definition of intervention and comparator, total number of participants in the intervention and comparator group, number of events in intervention and comparator group, study design, adjusted relative risk (RR) if reported. Each study was extracted by two review authors. Extracted data were compared and consensus was reached. Each study was assessed for limitations and graded according to the CHERG adaptation of the GRADE technique [15]. The basic grading system for CHERG is that randomized trials receive an initial grade of “high”; observational studies receive an initial grade of “low”; evidence generated through other designs receives an initial grade of “very low”. Adjustment of study quality is based on overall quality of study methods and execution (eg, adjustment for all plausible confounders). A detailed description of the grading system is available in Table S2 in the **Online Supplementary Document**.

Studies provided data on the effect of antibiotic treatment (penicillin, erythromycin, and azithromycin) on incidence of preterm births, stillbirths, low birthweight, perinatal death, and APO were included in the meta-analyses. For treatment of syphilis, we planned to perform three subgroup analyses based on comparisons conducted in the studies: adequate treatment (at least two doses of 2.4 million units penicillin) vs inadequate treatment; early treatment (in first or second trimester) vs late treatment; any treatment (at least one dose of 2.4 million units penicillin) vs no treatment. For treatment of chlamydia, we planned to perform two subgroup analyses based on comparisons conducted in the studies: treated successfully (treated with erythromycin or azithromycin and cured) vs treated but persistent or reinfected; treated (treated with erythromycin or azithromycin regardless of following infection status) vs untreated.

Meta-analyses were conducted with STATA version 17.0 statistical software. Due to the presence of heterogeneity, random effects models were used for all the analyses. Unadjusted RR were used because not enough studies provided adjusted RR. Unadjusted RR from each study was calculated based on total number of participants and number of events in each group. And corresponding 95% confidence intervals (CI) were reported. I square statistic for each outcome were also reported for assessment of heterogeneity across studies, where a value between 0 to 40 is considered low, 30 to 60 is considered moderate, 50 to 90 is deemed substantial, and 75-100 is deemed considerable.

## RESULTS

**Figure 1:**
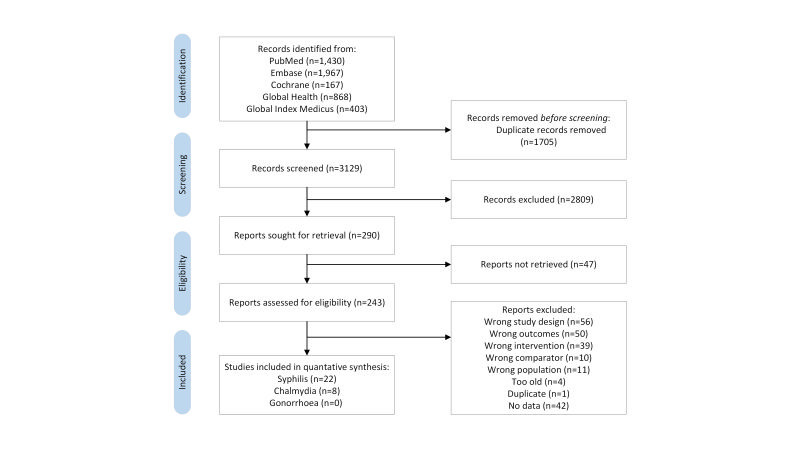
Flow diagram showing identification of studies. Some studies report on more than one outcome.

The search strategy from electronic database yielded 3143 records (**Figure 1**). After initial screening of the title or abstract we reviewed 290 full text articles. Thirty papers were abstracted and included in the present meta-analysis.

### Syphilis

Twenty-two observational studies conducted in developing countries were included for detection and treatment for active syphilis in pregnancy. There were 15 studies from China [16-30], three studies from Brazil [31-33], two studies from South Africa [34,35], one study from Russian [36], and one study with two cohorts paired in setting and time from Tanzania [37,38]. Sixteen studies [16-19,21-29,33,35,36] were retrospective, four studies [20,30,34,37,38] were prospective, and two studies [31,32] were cross-sectional. Only five studies [16,19,22,29,31] controlled potential confounders. The commonly adjusted covariates were maternal age, gravidity, marital status, maternal education, and attendance to antenatal care. As described above, unadjusted RR were used in the analysis. The CHERG Rules for Evidence Review were applied (**Table 1**).

**Table 1:** Quality assessment of overall evidence for effect of syphilis treatment in reducing suboptimal birth outcomes in developing countries

	Quality assessment					Summary of findings				
				Directness		Treated women with active syphilis		Untreated women with active syphilis*		
No of studies	Study design	Limitations	Consistency	Generalizability to population of interest	Generalizability to intervention of interest	No of events	No of births†	No of events	No of births†	RR (95% CI)‡
Outcome: preterm birth; quality of evidence: low										
15	Observational studies	No controlling for potential confounding variables	*I*^2^ = 67%	Maybe (10 studies from China)	Yes (all studies used penicillin)	593	7618	574	3425	0.48 (0.39-0.58)
Outcome: stillbirth; quality of evidence: low										
8	Observational studies	No controlling for potential confounding variables	*I^2^* = 66%	Maybe (6 studies from China)	Yes (all studies used penicillin)	84	9305	290	5362	0.21 (0.12-0.35)
Outcome: low birth weight; quality of evidence: moderate										
7	Observational studies	No controlling for potential confounding variables	*I*^2^ = 45%	Yes (all studies in LMICs)	Yes (all studies used penicillin)	391	6971	354	2807	0.50 (0.42-0.59)
Outcome: perinatal deaths; quality of evidence: low										
4	Observational studies	No controlling for potential confounding variables	*I*^2^ = 82%	Maybe (only studies from China and South Africa)	Yes (all studies used penicillin)	110	4232	190	1193	0.39 (0.20-0.76)
Outcome: adverse pregnancy outcomes; quality of evidence: very low										
3	Observational studies	No controlling for potential confounding variables	*I*^2^ = 98%	Maybe (all studies from China)	Yes (all studies used penicillin)	5063	52 160	3800	23 075	0.63 (0.49-0.81)

RR – relative risk, CI – confidence interval

*Untreated women also included women who received inadequate or delayed treatment.

†For studies that provided all three comparisons, we assumed the treated vs not treated group included participants in the adequate vs inadequate and early vs late groups.

‡Based on calculated pooled estimate using random effects model.

#### Evidence for the effectiveness of detection and treatment of active syphilis in pregnancy in reducing preterm births

**Figure 2:**
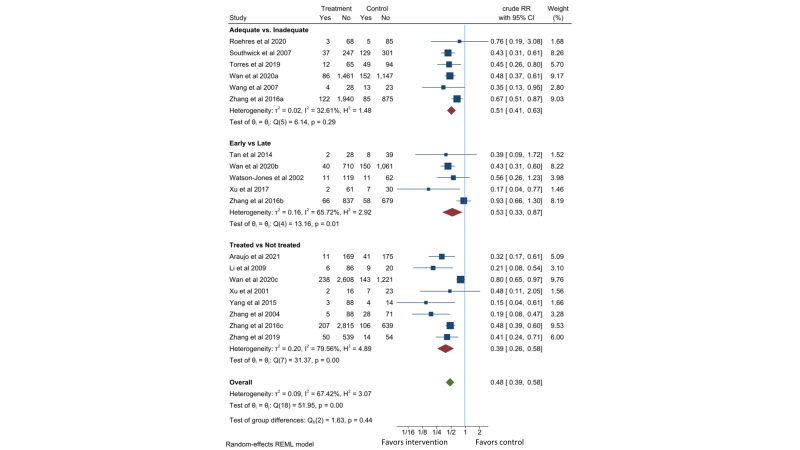
Effect of penicillin on preterm birth in pregnant women with active syphilis. The adequate group received at least two doses of 2.4MU penicillin. The early group received treatment in the first or second trimester. The treated group received at least one dose of 2.4MU penicillin.

Fifteen observational studies [17,21-29,31-33,36], including two Tanzania cohorts paired in setting and time [37,38], were identified. Compared to the pregnant women with active syphilis who received less than two dose of 2.4-million-unit penicillin or equivalent treatment, those who received two or more dose of penicillin treatment had 49% lower risk of preterm births (RR = 0.51, 95% CI = 0.41-0.63, *I*^2^ = 33%; six studies). Compared to delayed detection and treatment of active syphilis received in third trimester or at delivery, early detection during first or second trimester reduced the risk of preterm births by 47% (RR = 0.53, 95% CI = 0.33-0.87, *I*^2^ = 66%; five studies). Compared to pregnant women with active syphilis who did not receive any treatment, those who received at least one dose of 2.4-million-unit penicillin or equivalent treatment had 61% lower risk of preterm births (RR = 0.39, 95% CI = 0.26-0.58, *I*^2^ = 80%; eight studies). Two studies [22,29] contributed to all three comparisons. Combining these comparisons gave a RR of 0.48 (95% CI = 0.39-0.58, *I*^2^ = 67%) for preterm birth with penicillin treatment (**Figure 2**).

Fifteen observational cohort studies consistently reported reductions in preterm births of 52% (95% CI = 42%-61%) with penicillin treatment. Since none of these studies adequately controlled for potential confounding factors, other factors may have contributed to the effect. A majority of the studies were from China which limited the generalizability of the effect. The quality of evidence is considered low (**Table 1**).

#### Evidence for the effectiveness of detection and treatment of active syphilis in pregnancy in reducing stillbirths

**Figure 3:**
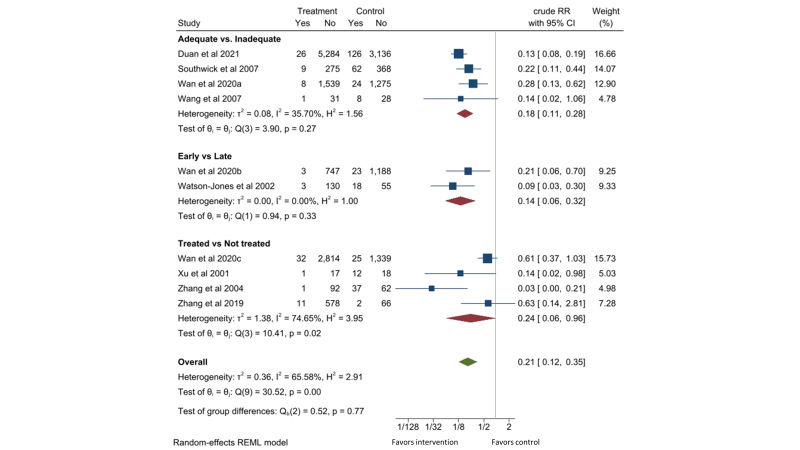
Effect of penicillin on stillbirth in pregnant women with active syphilis. The adequate group received at least two doses of 2.4MU penicillin. The early group received treatment in the first or second trimester. The treated group received at least one dose of 2.4MU penicillin.

Eight observational studies [16,22-24,27,29,36], including two Tanzania cohorts paired in setting and time [37,38], were retrieved. Compared to the pregnant women with active syphilis who received less than two dose of 2.4-million-unit penicillin or equivalent treatment, those who received two or more dose of penicillin treatment had 82% lower risk of stillbirths (RR = 0.18, 95% CI = 0.11-0.28, *I*^2^ = 36%; four studies). Compared to delayed detection and treatment of active syphilis received in third trimester or at delivery, early detection during first or second trimester reduced the risk of stillbirths by 86% (RR = 0.14, 95% CI = 0.06-0.32, *I*^2^ = 0%; two studies). Compared to pregnant women with active syphilis who did not receive any treatment, those who received at least one dose of 2.4-million-unit penicillin or equivalent treatment had 76% lower risk of stillbirths (RR = 0.24, 95% CI = 0.06-0.96, *I*^2^ = 75%; four studies). One study [22] contributed to all three comparisons. Combining these comparisons gave a RR of 0.21 (95% CI = 0.12-0.35, *I*^2^ = 66%) for stillbirths with penicillin treatment (**Figure 3**).

Eight observational cohort studies which did not control potential confounders in this analysis consistently reported reduction in stillbirths of 79% (95% CI = 65%-88%) with penicillin treatment. There were a sufficient number of participants and events, but the majority of the studies were from China which limited the generalizability of the effect. The quality of evidence is considered low (**Table 1**).

#### Evidence for the effectiveness of detection and treatment of active syphilis in pregnancy in reducing low birth weight

**Figure 4:**
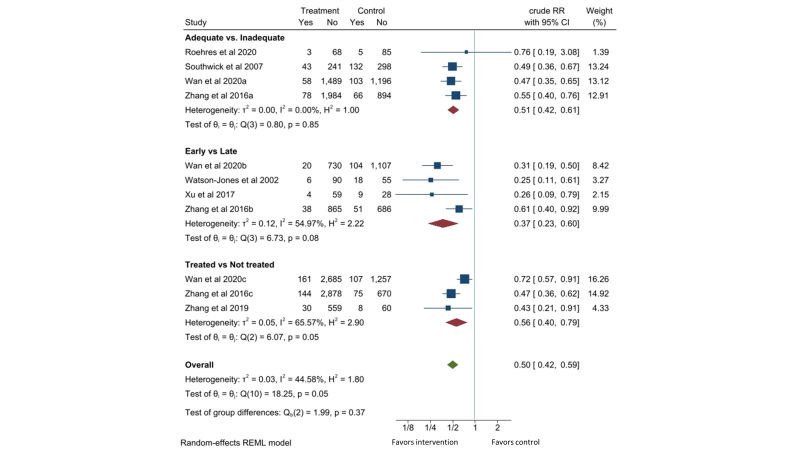
Effect of penicillin on low birth weight in pregnant women with active syphilis. The adequate group received at least two doses of 2.4MU penicillin. The early group received treatment in the first or second trimester. The treated group received at least one dose of 2.4MU penicillin.

Seven observational studies [22,25,28,29,32,36], including two Tanzania cohorts paired in setting and time [37,38], were identified. Compared to the pregnant women with active syphilis who received less than two dose of 2.4-million-unit penicillin or equivalent treatment, those who received two or more dose of penicillin treatment had 49% lower risk of low birth weight (RR = 0.51, 95% CI = 0.42-0.61, *I*^2^ = 0%; four studies). Compared to delayed detection and treatment of active syphilis received in third trimester or at delivery, early detection during first or second trimester reduced the risk of low birth weight by 63% (RR = 0.37, 95% CI = 0.23-0.60, *I*^2^ = 55%; four studies). Compared to pregnant women with active syphilis who did not receive any treatment, those who received at least one dose of 2.4-million-unit penicillin or equivalent treatment had 44% lower risk of low birth weight (RR = 0.56, 95% CI = 0.40-0.79, *I*^2^ = 66%; three studies). Two studies [22,29] contributed to all three comparisons. Combining these comparisons gave a RR of 0.50 (95% CI = 0.42-0.59, *I*^2^ = 45%) for low birth weight with penicillin treatment (**Figure 4**).

Seven observational cohort studies which did not control potential confounders in this analysis reported reduction in low birth weight of 50% (95% CI = 41%-58%) with penicillin treatment. Six out of seven studies found effects of penicillin treatment on reducing low birthweight. All the studies were conducted among population of interest, in LMICs. The quality of evidence is considered moderate (**Table 1**).

#### Evidence for the effectiveness of detection and treatment of active syphilis in pregnancy in reducing perinatal death

**Figure 5:**
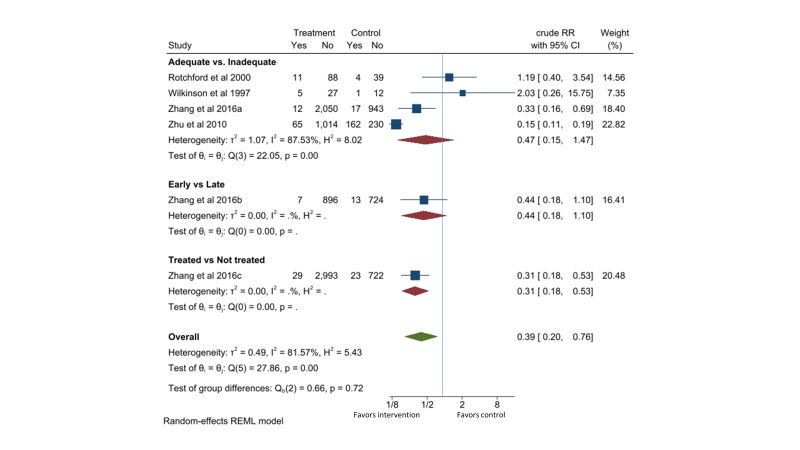
Effect of penicillin on perinatal death in pregnant women with active syphilis. The adequate group received at least two doses of 2.4MU penicillin. The early group received treatment in the first or second trimester. The treated group received at least one dose of 2.4MU penicillin.

Four observational studies were retrieved [28,30,34,35]. Compared to the pregnant women with active syphilis who received less than two dose of 2.4-million-unit penicillin or equivalent treatment, those who received two or more dose of penicillin treatment might have 53% lower risk of perinatal death (RR = 0.47, 95% CI 0.15-1.47, *I*^2^ = 88%; four studies). One study in China [28] compared early detection during first or second trimester with delayed detection and treatment of active syphilis received in third trimester or at delivery and found a RR of 0.44 (95% CI = 0.18-1.10) for perinatal death. The same study [28] also compared pregnant women with active syphilis who received at least one dose of 2.4-million-unit penicillin or equivalent treatment with those who did not receive any treatment and found a RR of 0.31 (95% CI = 0.18-0.53; one study) for perinatal death. Combining these comparisons gave a RR of 0.39 (95% CI = 0.20-0.76, *I*^2^ = 82%) for perinatal death with penicillin treatment (**Figure 5**).

Four observational cohort studies which did not control potential confounders in this analysis reported reduction in perinatal death of 61% (95% CI = 24%-80%) with penicillin treatment. Smaller number of participants were included in the analysis and only studies from China and South Africa were available. The quality of evidence is considered very low (**Table 1**).

#### Evidence for the effectiveness of detection and treatment of active syphilis in pregnancy in reducing any adverse pregnancy outcomes

**Figure 6:**
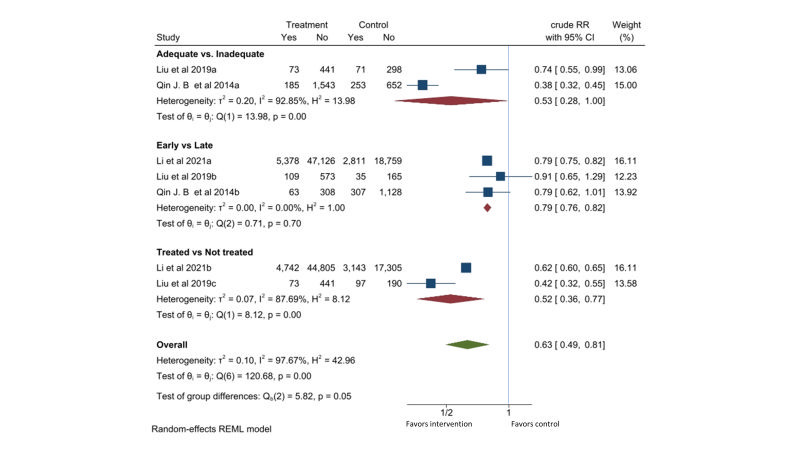
Effect of penicillin on adverse pregnancy outcomes (APO) in pregnant women with active syphilis. The adequate group received at least two doses of 2.4MU penicillin. The early group received treatment in the first or second trimester. The treated group received at least one dose of 2.4MU penicillin. APO any adverse pregnancy outcomes (one or more of the following outcomes: preterm, stillbirth, low birth weight, or perinatal death)

Three observational studies were identified [18-20]. Compared to the pregnant women with active syphilis who received less than two dose of 2.4-million-unit penicillin or equivalent treatment, those who received two or more dose of penicillin treatment had 47% lower risk of APO (RR = 0.53, 95% CI = 0.28-1.00, *I*^2^ = 93%; two studies). Compared to delayed detection and treatment of active syphilis received in third trimester or at delivery, early detection during first or second trimester reduced the risk of APO by 21% (RR = 0.79, 95% CI = 0.76-0.82, *I*^2^ = 0%; three studies). Compared to pregnant women with active syphilis who did not receive any treatment, those who received at least one dose of 2.4-million-unit penicillin or equivalent treatment had 48% lower risk of APO (RR = 0.52, 95% CI = 0.36-0.77, *I*^2^ = 88%; two studies). One study [19] contributed to all three comparisons. Two studies [18,20] contributed to two comparisons. Combining these comparisons gave a RR of 0.63 (95% CI = 0.49-0.81, *I*^2^ = 88%) for APO with penicillin treatment (**Figure 6**).

Three observational cohort studies reported reduction in APO of 37% (95% CI = 19%-51%) with penicillin treatment. All studies were from China, and none controlled for other potential confounders in their analysis. The quality of evidence is considered very low (**Table 1**).

### Chlamydia

Five observational studies [39-43] and two randomized control trials [44,45] were included for detection and treatment of chlamydia infection. There were five studies from United States [39-41,43,45], one study from Japan [42], and one study from India [44]. All the observational studies were retrospective and only one observational study provided adjusted RR [40]. As described above, unadjusted RRs were used in the meta-analysis. No study looked at stillbirths, perinatal deaths, or APO. Because chlamydia is prone to reinfection, some studies included repeated testing after treatment and had data on the individuals who did not respond to the treatment or were reinfected with chlamydia. The CHERG Rules for Evidence Review were applied (**Table 2**).

**Table 2:** Quality assessment of overall evidence for effect of chlamydia treatment in reducing suboptimal birth outcomes in developing countries

	Quality assessment					Summary of findings				
				Directness		Treated women with active syphilis		Untreated women with active syphilis*		
No of studies	Study design	Limitations	Consistency	Generalizability to population of interest	Generalizability to intervention of interest	No of events	No of births	No of events	No of births	RR (95% CI)†
Outcome: preterm birth; quality of evidence: low										
7	RCTs and observational studies	No controlling for potential confounding variables	*I*^2^ = 80%	No (6 studies from developed countries)	Yes (all studies used erythromycin or azithromycin)	297	3305	279	2163	0.58 (0.36-0.93)
Outcome: low birth weight; quality of evidence: low										
4	RCTs and observational studies	No controlling for potential confounding variables	*I*^2^ = 77%	No (3 studies from developed countries	Yes (all studies used erythromycin or azithromycin)	335	2918	257	1766	0.60 (0.36-1.00)

RCTs – randomized control trials, RR – relative risk, CI – confidence interval

*Untreated women also included women who received treatment but had persistent or recurrent chlamydia.

†Based on calculated pooled estimate using random effects model.

#### Evidence for the effectiveness of detection and treatment of chlamydia in pregnancy in reducing preterm births

**Figure 7:**
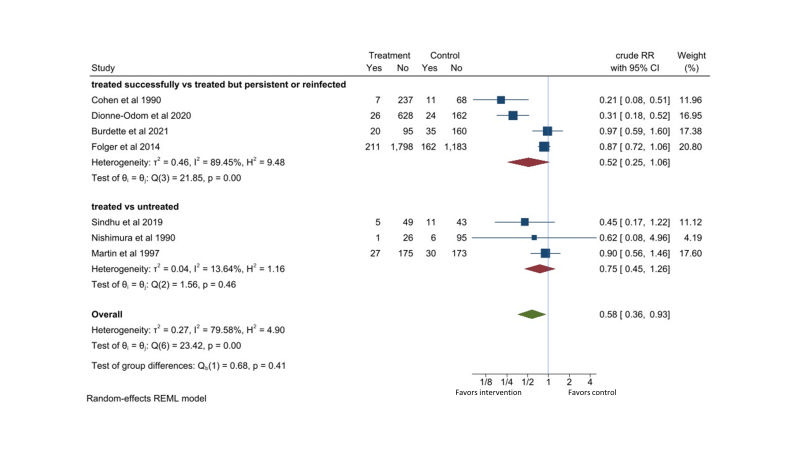
Effect of erythromycin or azithromycin on preterm birth in pregnant women with chlamydia infection.

Seven studies were retrieved [39-45]. Among pregnant women with chlamydia infection who received erythromycin or azithromycin, there was no significant differences in risk for preterm births (RR = 0.52, 95% CI = 0.25-1.06, *I*^2^ = 89%; four studies) between those with cleared chlamydia and those who had persistent or recurrent chlamydia [39-41,43]. There were also no significant differences in risk for preterm births (RR = 0.75, 95% CI = 0.45-1.26, *I*^2^ = 14%, three studies) between pregnant women with chlamydia who received erythromycin or azithromycin regardless of infection status after treatment and pregnant women with chlamydia who did not receive any treatment [42,44,45]. Combining these comparisons gave a RR of 0.58 (95% CI = 0.36-0.93, *I*^2^ = 80%) for preterm births with erythromycin or azithromycin treatment for chlamydia infected pregnancy (**Figure 7**).

Five observational cohort studies and two randomized control trials reported reduction in preterm birth of 42% (95% CI = 7%-64%) with erythromycin or azithromycin treatment. None of the observational studies controlled for potential confounding variables in this analysis. Six out of seven studies were conducted in developed countries. The quality of the evidence is considered low (**Table 2**).

#### Evidence for the effectiveness of detection and treatment of chlamydia in pregnancy in reducing low birth weight

**Figure 8:**
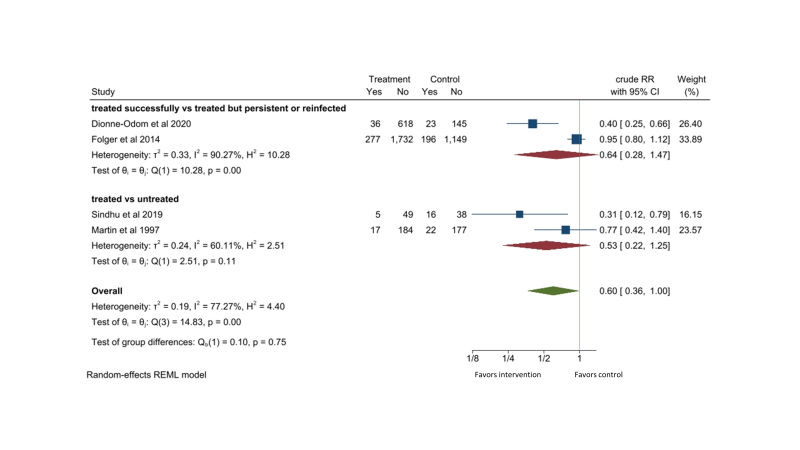
Effect of erythromycin or azithromycin on low birth weight in pregnant women with chlamydia infection.

Four studies were identified [39,40,44,45]. Among pregnant women with chlamydia infection who received erythromycin or azithromycin, there was no significant differences in risk for low birth weight (RR = 0.64, 95% CI = 0.28-1.47, *I*^2^ = 90%; two studies) between those with cleared chlamydia and those who had persistent or recurrent chlamydia [39,40]. There were also no significant differences in risk for low birth weight (RR = 0.53, 95% CI = 0.22-1.25, *I*^2^ = 60%; two studies) between pregnant women with chlamydia who received erythromycin or azithromycin regardless of infection status after treatment and pregnant women with chlamydia who did not receive any treatment [44,45]. Combining these comparisons gave a RR of 0.60 (95% CI = 0.36-1.00, *I*^2^ = 77%) for low birth weight with erythromycin or azithromycin treatment for chlamydia infected pregnancy (**Figure 8**).

Two observational cohort studies and two randomized control trials reported potential reduction in low birth weight of 40% (95% CI = 0%-64%) with erythromycin or azithromycin treatment. None of the observational studies controlled for potential confounding variables in their analysis. Three out of four studies were conducted in developed countries. The quality of the evidence is considered low (**Table 2**).

### Gonorrhoea

A limited number of papers studied the effect of treatment on suboptimal birth outcomes in pregnant women with gonorrhoea infection. Therefore, we did not identify any gonorrhoea studies that provided sufficient data to conduct a meta-analysis.

## DISCUSSION

The systematic review attempted to estimate the effect sizes of treatment for three common curable STIs on birth outcomes. We estimated that at least 2.4MU penicillin treatment for pregnant women with active syphilis reduced the risk of preterm birth by 52% (95% CI = 42%-61%; 11 043 participants, 15 studies; low quality); stillbirth by 79% (95% CI = 65%-88%; 14 667 participants, eight studies; low quality); and low birth weight by 50% (95% CI = 41%-58%; 9778 participants, seven studies; moderate quality). We also estimated that erythromycin or azithromycin treatment for pregnant women with chlamydia infection reduced the risk of preterm birth by 42% (95% CI = 7%-64%; 5468 participants, seven studies; low quality) and might reduce the risk of low birth weight by 40% (95% CI = 0%-64%; 4684 participants, four studies; low quality). No studies provided data on treatment of gonorrhoea, and therefore, no meta-analysis was conducted. All pooled estimates were based on a random effects model. Fixed effects models, not presented here, were also conducted but did not change the conclusion.

A 2011 systematic review found that penicillin treatment in pregnant women with active syphilis reduced the risk of stillbirth by 82% (67%-90%) [46]. The results were consistent with our finding of 79% (65%-88%) reduction in stillbirths. Conclusions on preterm birth were different as expected because a number of newer studies published after 2011 were included in this analysis, where we found a smaller reduction (52%, 42%-61%) than the previous review (64%, 53%-73%).

The Cochrane review on interventions for treating chlamydia infection in pregnancy only included randomized control trial. Therefore, only one study was identified, and the study did not find significant reduction in the risk of preterm births with treatment for chlamydia infection [47]. Our analysis included one more recent randomized trial and five observational studies, therefore our conclusion was slightly different, where we found a 42% (7%-64%) reduction in risk of preterm births with treatment for chlamydia infection in pregnancy.

There is no high-quality study on syphilis treatment because randomized controlled trials of effect of penicillin compared to no treatment would not be ethical. In this case, the evidence from observational studies is the best available data. Although most of the quality of evidence on penicillin treatment were low because potential confounding factors were not controlled in this analysis, the strength and the consistency of the associations makes it rather unlikely that the associations can be entirely explained by confounding factors. Consistency was observed in the subgroup analysis (adequate vs inadequate; early vs late; and treated vs not treated). We included these subgroup analyses 1) to capture as much data as possible 2) to understand the most effective treatment standard and to use the standard as the definition of the intervention included in the LiST model.

Another limitation for evidence around syphilis is that majority of the studies were conducted in China, which are not representative of LMICs. This limitation on generalizability to population of interest was considered during the quality assessment.

It is important to note that the strength of the recommendation is different from the quality of evidence. Detection and treatment of syphilis is an intervention which should clearly be strongly recommended given the biological plausibility, consistent and large effects, and the fact that addition of a new study will not change the beneficial effects.

We also feel that it is important to include in the LiST model interventions that are part of the standard of care even if the data on the specific value for efficacy are weak. For all three interventions we reviewed there are data on efficacy of the antibiotics used to treat syphilis, chlamydia, and gonorrhoea, as well as data on the risk associated with birth outcomes for untreated infections during pregnancy [13,14]. For countries where prevalence of one or more of these infections is high among pregnant women, not including these interventions because of lack of better data could lead to incorrect programming decisions that affect the health of women and their newborn children.

## CONCLUSIONS

Based on the pooled estimates from the meta-analysis and the quality of the evidence, we decided that in the LiST model, timely detection and treatment of syphilis during first or second trimester is estimated to reduce the risk of preterm births among pregnant women with active syphilis by 52% (42%-61%). The intervention is also estimated to reduce the risk of stillbirth among pregnant women with active syphilis by 79% (65%-88%).

We decided not to include chlamydia treatment in the model because the quality of evidence on efficacy is low, and the conclusions were not consistent across studies. No conclusion was available for treatment of gonorrhoea infection due to limited evidence. Higher-quality research is needed to ascertain efficacy of antibiotic treatment for chlamydia and gonorrhoea infections in pregnancy.

## Additional material


Online Supplementary Document
