# Supplementary material for: The impact of antibiotic treatment for syphilis, chlamydia, and gonorrhoea during pregnancy on birth outcomes: A systematic review and meta-analysis
Source: J Glob Health. 2023 Jun 16;13:04058. doi: 10.7189/jogh.13.04058 (PMC10273026; doi:10.7189/jogh.13.04058)
Supplement: Online Supplementary Document [file jogh-13-04058-s001.pdf]

Supplementary Table S1. Search Strategies

Database	Search Strategies
Pubmed	<u>Concept 1:</u><br>"Chlamydia Infections"[Mesh] OR "Gonorrhea"[Mesh] OR "Syphilis"[Mesh] OR chlamydia*[tw] OR chlamydiosis[tw] OR chlamydial[tw] OR gonorrhea*[tw] OR "gonococcal infection*"[tw] OR gonococcosis[tw] OR "gonococcus infection*"[tw] OR gonorrhoea*[tw] OR syphilis*[tw] OR syphilitic[tw]<br><br><u>Concept 2:</u><br>"Pregnancy"[Mesh] OR "Pregnant Women"[Mesh] OR "Pregnancy Trimesters"[Mesh] OR "Infectious Disease Transmission, Vertical"[Mesh] OR pregnan*[tw] OR trimester*[tw] OR "maternal transmission*"[tw] OR "mother to child transmission*"[tw] OR "maternal fetal transmission*"[tw] OR "transmission at birth"[tw] OR "maternal fetal exchange*"[tw]<br><br><u>Concept 3: (</u>birth outcomes (small for gestational age, low-birth weight, preterm birth)<br><br>"Premature Birth"[Mesh] OR "Obstetric Labor, Premature"[Mesh] OR "Infant, Premature"[Mesh] OR "Infant Mortality"[Mesh] OR "Infant, Low Birth Weight"[Mesh] OR "Infant, Very Low Birth Weight"[Mesh] OR "Fetal Growth Retardation"[Mesh] OR "Fetal Death"[Mesh] OR "Perinatal Death"[Mesh] OR "Perinatal Mortality"[Mesh]<br><br>OR "low birth weight*"[tw] OR "low birthweight*"[tw] OR "lower birth weight*"[tw] OR "lower birthweight*"[tw] OR VLBW[tw] OR ELBW[tw] OR LBW[tw] OR "fetal growth retard*"[tw] OR "intrauterine growth retard*"[tw] OR "intra uterine growth retard*"[tw] OR "fetal growth restrict*"[tw] OR "intrauterine growth restrict*"[tw] OR "intra uterine growth restrict*"[tw] OR IUGR[tw] OR "small for gestat*"[tw] OR SGA[tw] OR "small for date infant*"[tw] OR "fetal growth disorder*"[tw] OR "fetus growth disorder*"[tw] OR "fetus growth retard*"[tw] OR "growth retardation in utero"[tw] OR "in utero growth retard*"[tw] OR " in utero growth restriction"[tw] OR "growth restriction in utero"[tw] OR "prenatal growth retard*"[tw] OR "retarded intrauterine growth"[tw] OR "retarded intra uterine growth"[tw]<br><br>OR "premature birth*"[tw] OR "pre mature birth*"[tw] OR "preterm birth*"[tw] OR "pre term birth*"[tw] OR "premature labor*"[tw] OR "pre mature labor*"[tw] OR "preterm labor*"[tw] OR "pre term labor*"[tw] OR "premature labour*"[tw] OR "pre mature labour*"[tw] OR "preterm labour*"[tw] OR "pre term labour*"[tw] OR "premature obstetric labor*"[tw] OR "pre mature obstetric labor*"[tw] OR "preterm obstetric labor*"[tw] OR "pre term obstetric labor*"[tw] OR "premature obstetric labour*"[tw] OR "pre mature obstetric labour*"[tw] OR "preterm obstetric labour*"[tw] OR "pre term obstetric labour*"[tw] OR preemie*[tw] OR premie[tw] OR premies[tw] OR "premature deliver*"[tw] OR "pre mature deliver*"[tw] OR "preterm deliver*"[tw] OR "pre term deliver*"[tw] OR prematurity[tw] OR "early labor"[tw] OR "early labour"[tw]<br><br>OR stillbirth*[tw] OR "still birth*"[tw] OR "fetal death*"[tw] OR "fetal demise*"[tw] OR "fetal mortalit*"[tw] OR "perinatal death*"[tw] OR "perinatal demise*"[tw] OR "perinatal mortalit*"[tw] OR "peri natal death*"[tw] OR "peri natal demise*"[tw] OR "peri natal mortalit*"[tw] OR "intrauterine death*"[tw] OR "intra uterine death*"[tw] OR "prenatal death*"[tw] OR "pre natal death*"[tw] OR "prenatal demise*"[tw] OR "pre natal demise*"[tw] OR "prenatal mortalit*"[tw] OR "pre natal mortalit*"[tw] OR "newborn death*"[tw] OR "newborn demise*"[tw] OR "newborn mortalit*"[tw] OR "neonatal death*"[tw] OR "neonatal demis*"[tw] OR "neonatal mortalit*"[tw] OR IUFD[tw] OR "second trimester miscarriage*"[tw] OR "second trimester loss"[tw] OR "birth outcome*"[tw] OR "fetal outcome*"[tw] OR "pregnancy outcome*"[tw] OR "obstetric outcome*"[tw] OR "dead fetus"[tw] OR "fetus death"[tw]
Embase	<u>Concept 1:</u><br>'chlamydiasis'/exp OR 'gonorrhea'/exp OR 'syphilis'/exp OR (chlamydia* OR chlamydiosis OR chlamydial OR gonorrhea* OR "gonococcal infection*" OR gonococcosis OR "gonococcus infection*" OR gonorrhoea* OR syphilis* OR syphilitic):ab,ti,kw<br><br><u>Concept 2:</u><br>'pregnancy'/exp OR 'pregnant woman'/exp OR (pregnan* OR trimester* OR "maternal transmission*" OR "mother to child transmission*" OR "transmission at birth" OR "maternal fetal exchange*"):ab,ti,kw<br><br><u>Concept 3:</u><br>'prematurity'/exp OR 'premature labor'/exp OR 'fetus mortality'/exp OR 'perinatal mortality'/exp OR 'premature mortality'/exp OR 'prenatal mortality'/exp OR 'low birth weight'/exp OR 'intrauterine growth retardation'/exp OR 'perinatal death'/exp OR 'newborn death'/exp<br><br>'low birth weight':ab,ti,kw OR 'low birth weights':ab,ti,kw OR 'low birthweight':ab,ti,kw OR 'low birthweights':ab,ti,kw OR 'lower birth weight':ab,ti,kw OR 'lower birth weights':ab,ti,kw OR 'lower birthweight':ab,ti,kw OR 'lower birthweights':ab,ti,kw OR vlbw:ab,ti,kw OR elbw:ab,ti,kw OR lbw:ab,ti,kw OR (('fetal growth' NEAR/2 retard*):ab,ti,kw) OR (('fetus growth' NEAR/2 retard*):ab,ti,kw) OR (('intrauterine growth' NEAR/2 retard*):ab,ti,kw) OR (('fetal growth' NEAR/2 restrict*):ab,ti,kw) OR (('fetus growth' NEAR/2 restrict*):ab,ti,kw) OR (('fetal growth' NEAR/2 disorder*):ab,ti,kw) OR (('fetus growth' NEAR/2 disorder*):ab,ti,kw) OR (('intrauterine growth' NEAR/2 restrict*):ab,ti,kw) OR iugr:ab,ti,kw OR

	((small NEAR/2 gestat*):ab,ti,kw) OR sga:ab,ti,kw OR (('small for date' NEAR/2 infant*):ab,ti,kw) OR (('prenatal growth' NEAR/2 retard*):ab,ti,kw) OR (('prenatal growth' NEAR/2 restrict*):ab,ti,kw) OR (('prenatal growth' NEAR/2 disorder*):ab,ti,kw) OR (('pre natal growth' NEAR/2 retard*):ab,ti,kw) OR (('pre natal growth' NEAR/2 restrict*):ab,ti,kw) OR (('pre natal growth' NEAR/2 disorder*):ab,ti,kw) OR (('growth retardation' NEAR/2 'in utero'):ab,ti,kw) OR (('in utero growth' NEAR/2 retard*):ab,ti,kw) OR (('in utero growth' NEAR/2 restriction*):ab,ti,kw) OR 'growth restriction in utero':ab,ti,kw OR 'retarded intra uterine growth':ab,ti,kw<br><br>(((prematur* OR 'pre mature' OR 'pre term' OR preterm) NEAR/2 (birth* OR labor* OR labour* OR delivery)):ab,ti,kw) OR preemie*:ab,ti,kw OR premie:ab,ti,kw OR premies:ab,ti,kw OR 'early labor':ab,ti,kw OR 'early labour':ab,ti,kw<br><br>stillbirth*:ab,ti,kw OR 'still birth':ab,ti,kw OR 'still births':ab,ti,kw OR iufd:ab,ti,kw OR 'second trimester miscarriage':ab,ti,kw OR 'second trimester miscarriages':ab,ti,kw OR 'second trimester loss':ab,ti,kw OR 'dead fetus':ab,ti,kw OR 'fetus death':ab,ti,kw<br><br>((fetal OR perinatal* OR 'peri natal*' OR intrauterine OR 'intra uterine' OR 'in utero' OR prenatal* OR 'pre natal*' OR newborn* OR neonat*) NEAR/2 (death* OR demise* OR mortalit*)):ab,ti,kw<br><br>((birth* OR fetal OR pregnanc* OR obstetric*) NEAR/2 outcome*):ab,ti,kw<br><br>#3 OR #4 OR #5 OR #6 OR #7<br><br>#1 AND #2 AND #8
Cochrane	#1 MeSH descriptor: [Chlamydia Infections] explode all trees<br>#2 MeSH descriptor: [Gonorrhea] explode all trees<br>#3 MeSH descriptor: [Syphilis] explode all trees<br>#4 chlamydia* OR chlamydiosis OR chlamydial OR gonorrhea* OR "gonococcal infection" OR "gonococcal infections" OR gonococcosis OR "gonococcus infection" OR "gonococcus infections" OR gonorrhoea* OR syphilis* OR syphilitic<br>#5 {OR #1-#4}<br>#6 MeSH descriptor: [Pregnancy] explode all trees<br>#7 MeSH descriptor: [Pregnant Women] explode all trees<br>#8 MeSH descriptor: [Pregnancy Trimesters] explode all trees<br>#9 MeSH descriptor: [Infectious Disease Transmission, Vertical] explode all trees<br>#10 pregnan* OR trimester* OR (maternal NEXT/1 transmission*) OR ("mother to child" NEXT/1 transmission*) OR "transmission at birth" OR ("maternal fetal" NEXT/1 exchange*)<br>#11 {OR #6-#10}<br>#12 MeSH descriptor: [Premature Birth] explode all trees<br>#13 MeSH descriptor: [Obstetric Labor, Premature] explode all trees<br>#14 MeSH descriptor: [Infant, Premature] explode all trees<br>#15 MeSH descriptor: [Infant Mortality] explode all trees<br>#16 MeSH descriptor: [Infant, Low Birth Weight] explode all trees<br>#17 MeSH descriptor: [Infant, Very Low Birth Weight] explode all trees<br>#18 MeSH descriptor: [Fetal Growth Retardation] explode all trees<br>#19 MeSH descriptor: [Fetal Death] explode all trees<br>#20 MeSH descriptor: [Perinatal Death] explode all trees<br>#21 "low birth weight" OR "low birth weights" OR "low birthweight" OR "low birthweights" OR "lower birth weight" OR "lower birth weights" OR "lower birthweight" OR "lower birthweights" OR VLBW OR ELBW OR LBW OR ("fetal growth" NEAR/2 retard*) OR ("fetus growth" NEAR/2 retard*) OR ("intrauterine growth" NEAR/2 retard*) OR ("fetal growth" NEAR/2 restrict*) OR ("fetus growth" NEAR/2 restrict*) OR ("fetal growth" NEAR/2 disorder*) OR ("fetus growth" NEAR/2 disorder*) OR ("intrauterine growth" NEAR/2 restrict*) OR IUGR OR (small NEAR/2 gestat*) OR SGA OR ("small for date" NEAR/2 infant*) OR ("prenatal growth" NEAR/2 retard*) OR ("prenatal growth" NEAR/2 restrict*) OR ("prenatal growth" NEAR/2 disorder*) OR ("pre natal growth" NEAR/2 retard*) OR ("pre natal growth" NEAR/2 restrict*) OR ("pre natal growth" NEAR/2 disorder*) OR ("growth retardation" NEAR/2 "in utero") OR ("in utero growth" NEAR/2 retard*) OR ("in utero growth" NEAR/2 restriction*) OR "growth restriction in utero" OR "retarded intra uterine growth"<br>#22 ((prematur* OR "pre mature" OR "pre term" OR preterm) NEAR/2 (birth* OR labor* OR labour* OR delivery)) OR preemie* OR premie OR premies OR "early labor" OR "early labour"<br>#23 stillbirth* OR "still birth" OR "still births" OR ((fetal OR perinatal* OR "peri natal" OR "peri natally" OR intrauterine OR "intra uterine" OR "in utero" OR prenat* OR "pre natal" OR "pre natally" OR newborn* OR neonat*) NEAR/2 (death* OR demise* OR mortalit*)) OR IUFD OR "second trimester miscarriage" OR "second trimester miscarriages" OR "second trimester loss" OR "dead fetus" OR "fetus death"<br>#24 (birth* OR fetal OR pregnanc* OR obstetric*) NEAR/2 (outcome*)<br>#25 {OR #12-#24}<br>#26 #5 AND #11 AND #25
Global Health	Concept 1:<br>exp Chlamydia/ or exp gonorrhoea/ or exp syphilis/ or (chlamydia* or chlamydiosis or chlamydial or gonorrhea* or "gonococcal infection*" or gonococcosis or "gonococcus infection*" or gonorrhoea* or syphilis* or syphilitic).tw.

	Concept 2:<br>exp pregnancy/ or exp pregnant women/ or exp vertical transmission/ or exp maternal transmission/ or exp maternal-fetal exchange/ or (pregnan* or trimester* or "maternal transmission*" or "mother to child transmission*" or "maternal fetal transmission*" or "transmission at birth" or "maternal fetal exchange*").tw.<br><br>Concept 3:<br>exp prematurity/ or exp premature infants/ or exp infant mortality/ or exp neonatal mortality/ or exp fetal death/ or exp stillbirths/ or exp perinatal mortality/ or exp low birth weight infants/ or exp fetal growth/ or exp growth retardation/<br><br>("low birth weight*" OR "low birthweight*" OR "lower birth weight*" OR "lower birthweight*" OR vlbw OR elbw OR lbw OR (("fetal growth" adj2 retard*)) OR (("fetus growth" adj2 retard*)) OR (("intrauterine growth" adj2 retard*)) OR (("fetal growth" adj2 restrict*)) OR (("fetus growth" adj2 restrict*)) OR (("fetal growth" adj2 disorder*)) OR (("fetus growth" adj2 disorder*)) OR (("intrauterine growth" adj2 restrict*)) OR iugr OR ((small adj2 gestat*)) OR sga OR (("small for date" adj2 infant*)) OR (("prenatal growth" adj2 retard*)) OR (("prenatal growth" adj2 restrict*)) OR (("prenatal growth" adj2 disorder*)) OR (("pre natal growth" adj2 retard*)) OR (("pre natal growth" adj2 restrict*)) OR (("pre natal growth" adj2 disorder*)) OR (("growth retardation" adj2 "in utero")) OR (("in utero growth" adj2 retard*)) OR (("in utero growth" adj2 restriction*)) OR "growth restriction in utero" OR "retarded intra uterine growth").tw.<br><br>(((prematur* OR "pre mature" OR "pre term" OR preterm) adj2 (birth* OR labor* OR labour* OR delivery)) OR preemie* OR premie OR premies OR "early labor" OR "early labour").tw.<br><br>(stillbirth* OR "still birth" OR "still births" OR (((fetal OR perinatal* OR "peri natal*" OR intrauterine OR "intra uterine" OR "in utero" OR newborn* OR prenat*) adj2 (death* OR demise* OR mortalit*))) OR iufd OR "second trimester miscarriage" OR "second trimester miscarriages" OR "second trimester loss" OR "dead fetus" OR "fetus death").tw.<br><br>((birth* OR fetal OR pregnanc* OR obstetric*) adj2 outcome*).tw.<br><br>3 or 4 or 5 or 6 or 7<br><br>1 and 2 and 8
Global Index Medicus	(MH: C01.150.252.400.210.125$ OR MH:C01.150.252.734.301$ OR MH:C01.221.812.281.301$ OR MH:C01.778.281.301$ OR MH:C12.294.668.281.301$ OR MH:C13.351.500.711.281.301$ OR chlamydia$ OR chlamydiosis OR chlamydial OR gonorrhea$ OR "gonococcal infection" OR "gonococcal infections" OR gonococcosis OR "gonococcus infection" OR "gonococcus infections" OR gonorrhoea$ OR syphilis$ OR syphilitic)<br>AND<br>(MH:G08.686.784.769$ OR MH:M01.975.807$ OR MH:SP3.001.004.080.010$ OR MH:G08.686.707$ OR MH:N06.850.335.875$ OR pregnan$ OR trimester$ OR "maternal transmission" OR "maternal transmissions" OR "mother to child transmission" OR "mother to child transmissions" OR "maternal fetal transmission" OR "maternal fetal transmissions" OR "transmission at birth" OR "maternal fetal exchange")<br>AND<br>MH:C13.703.420.491.500$ OR MH:C13.703.420.491$ OR MH:M01.060.703.520.520$ OR MH:E05.318.308.985.550.475$ OR MH:N01.224.935.698.489$ OR MH:N06.850.505.400.975.550.475$ OR MH:N06.850.520.308.985.550.475$ OR MH:SP3.076.187.173.164$ OR MH:SP4.127.413.629.905.376$ OR MH:SP5.006.052.168.154.110$ OR MH:M01.060.703.520.460$ OR MH:C13.703.277.370$ OR MH:C16.300.390$ OR MH:C23.550.393.450$ OR MH:C13.703.223$ OR MH:C23.550.260.585$ OR "low birth weight" OR "low birth weights" OR "low birthweight" OR "low birthweights" OR "lower birth weight" OR "lower birth weights" OR "lower birthweight" OR "lower birthweights" OR vlbw OR elbw OR lbw OR ("fetal growth" AND retard*) OR ("fetus growth" AND retard*) OR ("intrauterine growth" AND retard*) OR ("fetal growth" AND restrict*) OR ("fetus growth" AND restrict*) OR ("fetal growth" AND disorder*) OR ("fetus growth" AND disorder*) OR ("intrauterine growth" AND restrict*) OR iugr OR (small AND gestat*) OR sga OR ("small for date" AND infant*) OR ("prenatal growth" AND retard*) OR ("prenatal growth" AND restrict*) OR ("prenatal growth" AND disorder*) OR ("pre natal growth" AND retard*) OR ("pre natal growth" AND restrict*) OR ("pre natal growth" AND disorder*) OR ("growth retardation" AND "in utero") OR ("in utero growth" AND retard*) OR ("in utero growth" AND restriction*) OR "growth restriction in utero" OR "retarded intra uterine growth" OR ((prematur* OR "pre mature" OR "pre term" OR preterm) AND (birth* OR labor* OR labour* OR delivery)) OR preemie* OR premie OR premies OR "early labor" OR "early labour" OR stillbirth* OR "still birth" OR "still births" OR ((fetal OR perinatal OR intrauterine OR "intra uterine" OR "in utero" OR newborn* OR prenat*) AND (death* OR demise* OR mortalit*)) OR iufd OR "second trimester miscarriage" OR "second trimester miscarriages" OR "second trimester loss" OR "dead fetus" OR "fetus death" OR ((birth* OR fetal OR pregnanc* OR obstetric*) AND outcome*)

Supplementary Table S2. Grading system for study design and study quality used in the CHERG reviews of intervention effects.

Scoring system<br><br>Each study is assigned a single score based on a four-point continuum: 'high', 'moderate', 'low' or 'very low'.
Score for study design<br><br>Randomized trials receive an initial grade of 'high'; observational studies receive an initial grade of 'low'; evidence generated through other designs receives an initial grade of 'very low'.
Adjustment for study quality<br><br>Review groups conduct a careful assessment of study methods and execution, and adjust the initial score assigned for study design if necessary based on the rules listed below.<br><br>Rules for adjusting study design scores<br>• A score should be downgraded one level is there are serious questions with regard to the overall quality of the study methods or sample size, evidence or suspicion of reporting bias or inconsistencies with other data sources that raise doubts about the validity of the results.<br>• A score should be downgraded one level if there are very serious questions with regard to who the study was executed.<br>• A score should be upgraded one level if the researchers either controlled or accounted for all plausible confounders that would have reduced the effect of the intervention on the health outcomes.
